# Mortality of Chicago in 1874

**Published:** 1875-02-15

**Authors:** 


### MORTALITY OF CHICAGO IN 1874.

ACCORDING to the statistics kindly furnished us by the Sanitary Superintendent, the gross mortality of this city in the year 1874 was 8,025, being a fraction less than 1 in 50 of the population. Of the whole number of deaths 903 were in hospital and from accidents. The remaining 7,122 were distributed among the several wards as follows: First Ward, 34; Second, 48; Third, 253; Fourth, 151; Fifth, 250; Sixth, 751; Seventh, 783; Eighth, 627; Ninth, 532; Tenth, 190; Eleventh, 202; Twelfth, 239; Thirteenth, 223; Fourteenth, 238; Fifteenth, 1,088; Sixteenth, 513; Seventeenth, 418; Eighteenth, 405; Nineteenth, 57; Twentieth, 120. By comparing the deaths in the several wards with the population, we see most strikingly the effects of sanitary conditions on the ratio of mortality. Thus in the first, second, fourth, fifth, tenth, twelfth, thirteenth, fourteenth, nineteenth and twentieth wards the ratio of deaths to the population varied from 1 in 70¼ in the fourteenth and twentieth to 1 in 168⅓ in the first; while in the third, sixth, seventh, eighth, ninth, eleventh, fifteenth, sixteenth, seventeenth and eighteenth wards, the ratio varied from 1 in 40½ in the seventh to 1 in 69½ in the eleventh.

The first ten wards named contain the largest part of the native American population, and embraces the best improved and most completely sewered part of the city. The last ten are occupied mostly by the foreign-born and laboring classes, and embraces the Bridgeport and Stockyard region in the south-west, and the north and north-western parts of the city. The influence of temperature and season of the year on the ratio of mortality is seen in the following figures:

Number of deaths in all the wards, not including those from accidents and in public hospital, was in January, 494; February, 414; March, 453; April, 404; May, 434; June, 440; July, 1,343; August, 1,138; September, 732; October, 498; November, 385; December, 387. Only little less than half the number of deaths for the year took place during the months of July, August and September, the number in July being more than three times that of June. The great excess of mortality in the three months named was almost wholly among the children under three years of age.

